# Exposure of silver-nanoparticles and silver-ions to lung cells *in vitro* at the air-liquid interface

**DOI:** 10.1186/1743-8977-10-11

**Published:** 2013-04-04

**Authors:** Fabian Herzog, Martin JD Clift, Flavio Piccapietra, Renata Behra, Otmar Schmid, Alke Petri-Fink, Barbara Rothen-Rutishauser

**Affiliations:** 1 Adolphe Merkle Institute, Bio-Nanomaterials, University of Fribourg, Marly, Switzerland; 2 Eawag ,Swiss Federal Institute of Aquatic Science and Technology, Dübendorf, Switzerland; 3 Comprehensive Pneumology Center, Institute of Lung Biology and Disease Helmholtz Zentrum München, Neuherberg, Germany; 4 Department of Chemistry, University of Fribourg, Fribourg, Switzerland; 5 Respiratory Medicine, Department of Clinical Research, Inselspital University Hospital, University of Bern, Bern, Switzerland

## Abstract

**Background:**

Due to its antibacterial properties, silver (Ag) has been used in more consumer products than any other nanomaterial so far. Despite the promising advantages posed by using Ag-nanoparticles (NPs), their interaction with mammalian systems is currently not fully understood. An exposure route via inhalation is of primary concern for humans in an occupational setting. Aim of this study was therefore to investigate the potential adverse effects of aerosolised Ag-NPs using a human epithelial airway barrier model composed of A549, monocyte derived macrophage and dendritic cells cultured *in vitro* at the air-liquid interface. Cell cultures were exposed to 20 nm citrate-coated Ag-NPs with a deposition of 30 and 278 ng/cm^2^ respectively and incubated for 4 h and 24 h. To elucidate whether any effects of Ag-NPs are due to ionic effects, Ag-Nitrate (AgNO_3_) solutions were aerosolised at the same molecular mass concentrations.

**Results:**

Agglomerates of Ag-NPs were detected at 24 h post exposure in vesicular structures inside cells but the cellular integrity was not impaired upon Ag-NP exposures. Minimal cytotoxicity, by measuring the release of lactate dehydrogenase, could only be detected following a higher concentrated AgNO_3_-solution. A release of pro-inflammatory markers TNF-α and IL-8 was neither observed upon Ag-NP and AgNO_3_ exposures as well as was not affected when cells were pre-stimulated with lipopolysaccharide (LPS). Also, an induction of mRNA expression of *TNF-α* and *IL-8*, could only be observed for the highest AgNO_3_ concentration alone or even significantly increased when pre-stimulated with LPS after 4 h. However, this effect disappeared after 24 h. Furthermore, oxidative stress markers (*HMOX-1, SOD-1*) were expressed after 4 h in a concentration dependent manner following AgNO_3_ exposures only.

**Conclusions:**

With an experimental setup reflecting physiological exposure conditions in the human lung more realistic, the present study indicates that Ag-NPs do not cause adverse effects and cells were only sensitive to high Ag-ion concentrations. Chronic exposure scenarios however, are needed to reveal further insight into the fate of Ag-NPs after deposition and cell interactions.

## Background

Nanotechnology is a rapidly growing field, with the application of engineered nanomaterials in daily life constantly increasing. Nanoparticles (NPs) are defined by the European Commission as materials whose main constitutes have three dimension between 1 and 100 billionth of a metre [1]. Numerous different types of NPs, have been engineered for use in a wide array of consumer, industrial and technological applications due to their high surface to volume ratio that leads to unique physical and chemical properties. As a result of their widespread applications therefore, a significant increase of commercial nanotechnology industry is presumed within the next years [2]. So far, silver nanoparticles (Ag-NPs) have been used in more consumer products than any other nanomaterial [3], mainly due to its antimicrobial properties [4,5]. The use of Ag as an antimicrobial agent however, is not a new concept as it has been used for example since the 17th century as an essential multipurpose medicinal product [6]. Examples of recent consumer applications using Ag as antimicrobial agents consist of: food supplements, materials for food packaging, coatings on medical devices, water disinfectants, air filters, electronic appliances, odour-resistant textile fabrics and cosmetic products, such as deodorants [7,8].

Despite the promising advantages posed by using Ag-NPs in such applications, the possible health effects associated with the inevitable human exposure to NPs [9,10] has raised concerns as to the mass use and production of Ag-NPs without having a clear understanding of their specific interaction with biological systems [11]. Therefore, increased attention has been given to the potential human health and environmental effects following Ag-NP exposure [12]. In regards to the human interaction with Ag-NPs, numerous different exposure routes exist: via the lung, skin, bloodstream and ingestion. It is assumed, however, that inhalation of Ag-NPs is of primary concern for humans in an occupational setting [13].

It has been shown that following *in vitro* exposures of Ag-NPs to various different cell types in particular immune cells such as macrophages and monocytes [14-16] and epithelial lung cells [17-19] that these NPs can induce significant cytotoxicity and (pro)-inflammatory cytokine release as well as induce increased levels of oxidative stress and reactive oxygen species (ROS) production over acute time periods (≤48 hours) [17,20,21]. Furthermore, investigation into the potential genotoxicity of Ag-NPs has also shown that these NPs can cause significant DNA damage in human lung cells *in vitro*[20,22]. Despite this, a clear understanding of the specific Ag-NP cell-interaction is severely limited. This is highlighted by the disparity whether the just described effects of Ag-NPs are in fact a direct result of the NPs themselves, or rather due to the interaction with Ag-ions [23,24] that are released when Ag-NPs are placed into solution [25]. In the presence of moisture, metallic Ag-NPs oxidize, which results in the release of Ag-ions. Because Ag oxidation is a slow reaction, the size of Ag NPs is critical to achieve microorganism growth control [26]. Several studies illustrate this contradicting picture and further highlight other aspects that may contribute towards the biological impact of Ag. These include the shape and size [27,28], NP related ROS production [29] or a combined mechanism of particle and ion exposure [30]. Due to the contradicting literature and the unknown mechanistic behaviour of Ag toxicity, it is imperative therefore, that increased, in-depth research is performed in order to assess if the potential advantageous properties of Ag-NPs can be realised safely in commercial nanotechnological applications.

There are a number of different experimental approaches described in order to investigate the possible adverse effects of Ag-NPs towards the human lung. Many studies are performed *in vitro* using cultured lung cells under submerged conditions [31-34]. Such exposures however, do not represent the conditions that would be expected in the human lung when a NP containing aerosol is inhaled. In animals, NPs can be applied via instillation [35] or by inhalation [36]. Since there are many efforts on-going to use sophisticated *in vitro* methods for toxicology testing in order to reduce the number of invasive animal-based testing strategies [37] our research group has established and evaluated an *in vitro* model of the human epithelial airway barrier composed of epithelial cells and the two most important immune cells of the lung (*i.e.* macrophages and dendritic cells), to study NP lung-cell interactions and their possible responses [38]. Since this model can be used at the air-liquid interface it allows the direct exposure of cells to an aerosol [39], thus representing a realistic situation following inhalation of NPs. Recently, a novel dose controlled air-liquid interface cell exposure (ALICE) system for NP aerosols [40] has been established and has been employed to evaluate the possible adverse effects of zinc oxide [40] and gold NPs [41,42]. Therefore, the aim of the present study was to use the same experimental set-up to assess the cytotoxicity, the oxidative potential and pro-inflammatory effects of Ag-NPs in comparison to Ag-ions at the same molecular mass.

## Results

### Particle exposure and characterisation

**Figure 1:**
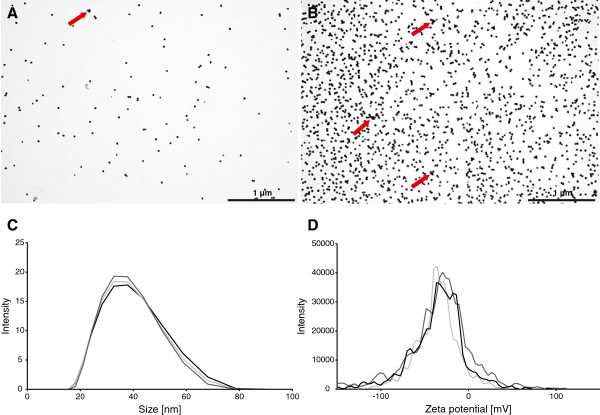
**Particle deposition in the ALICE, size characterization and stability.** Transmission electron microscopy (TEM) pictures of 1x (**A**) and 10× (**B**) concentrated 20 nm Ag NPs deposited onto TEM grids by nebulization with the ALICE system (scale bar = 1 μm). Agglomerates are indicated with red arrows. The size distribution (**C**) was measured by dynamic light scattering and showed an average hydrodynamic diameter of 33.4 ± 0.23 nm. The average zeta potential (**D**) was defined at -37.5 ± 0.25 mV.

Inductively coupled plasma mass spectrometry (ICP-MS) measurements showed that the stock solutions have an Ag concentration of 6 μg/mL. In order to use similar NP concentrations as the previous study done with gold NPs [41,42] the stock solutions were concentrated 4 and 40 times by ultrafiltration to receive two different concentrations of 24 and 240 μg/mL respectively. Dissolved Ag was determined to be 1.25 ± 0.05% and 0.12 ± 0.01%, respectively, of total Ag in Ag-NP suspensions. Nebulization in the air liquid exposure system was performed using 1 mL of each Ag-NP solution. Particle deposition was calculated by measuring the amount of Ag deposited in wells filled with 1 mL ddH_2_O with ICP-MS and revealed a deposition of 30 ± 6.6 ng/cm^2^ and 278 ± 43.6 ng/cm^2^ respectively. These findings correspond to a deposition efficiency of 50% and 47% respectively. The distribution and the state of agglomeration of the deposited NPs was qualitatively analysed with transmission electron microscopy (TEM) by particle exposure onto TEM grids (Figure 1A and B). The images show a homogeneous distribution of particles at both concentrations and only minor agglomeration of particles after nebulization (Figure 1; red arrows). The stock solution was analysed by dynamic light scattering (DLS) and laser doppler anemometry (LDA) with a Malvern Zetasizer (Zetasizer Nano Series, Malvern Instruments Ltd., Worcestershire, UK) to determine the hydrodynamic diameter and the zeta-potential. In Figure 1C the size distribution of the Ag-NPs is shown. The average hydrodynamic diameter measured was 33.4 ± 0.2 nm. The zeta potential (Figure 1D) was determined to be -37.5 ± 0.3 mV as the citrate-coating providing a negative charge. These data indicate that the Ag-NPs have a narrow size distribution and are monodisperse when applied to the cells.

### Lung cell morphology and intracellular Ag-NP localisation

**Figure 2:**
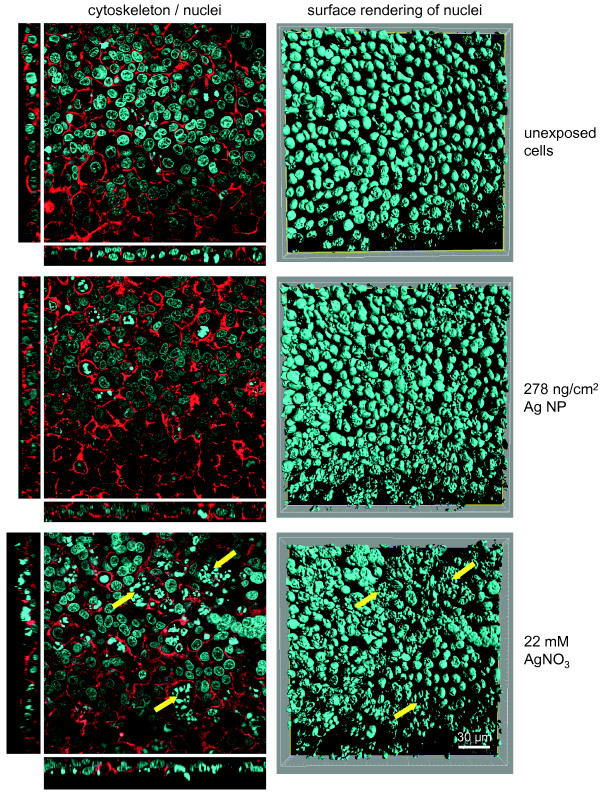
**Cell morphology of exposed cells.** Illustrated pictures represent unexposed, 278 ng/cm^2^ Ag-NP and 22 mM AgNO_3_ exposed triple cell co-cultures. At 24 h after exposure, the cells were fixed and stained for the actin cytoskeleton (phalloidin rhodamine; red) and DNA (DAPI; blue). Examples of morphological changes in form of augmented DNA condensation and alterations in the cytoskeleton were marked with yellow arrows. Images for the actin cytoskeleton and nuclei on the left side are represented as single optical xy projections with representative side views in xz (bottom) and yz (left) direction. Nuclei on the right side are visualized by surface rendering of xy stacks (scale bar = 30 μm).

A triple cell co-culture system composed of A549 epithelial cells combined with monocyte-derived macrophages (MDM) and dendritic cells (MDDC) cultured at the air-liquid interface [39,41] was used. After Ag-NP and AgNO_3_ exposure the cell morphology was studied by laser scanning microscopy (LSM) (Figure 2). The exposure of cells to Ag-NPs at both concentrations did not affect cell morphology if compared to untreated control. However, DNA condensation (Figure 2; yellow arrows) and alterations in the actin cytoskeleton were observed in cells exposed to 22 mM but not for 0.22 and 2.2 mM AgNO_3_ concentrations.

**Figure 3:**
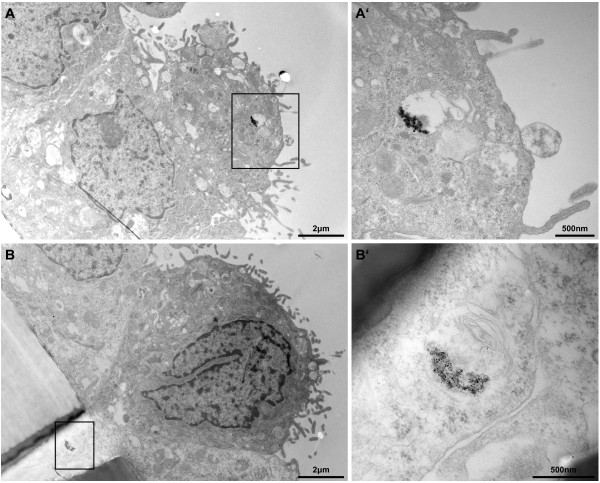
**Particle uptake in the upper transwell cell layer.** Ag-NPs were found in the upper cell layer of the transwell membrane (**A**) and in cells crossing the transwell insert (**B**) as aggregates inside vesicles at 24 h post-exposure (scale bar = 2 μm). Overall cell morphology upon Ag-NP exposure was similar to untreated control. **A’** and **B’** represent a higher magnification of the black marked box of the opposing picture (scale bar = 500 nm). **B’** reveals particle agglomerates inside a multilamellar body.

TEM was then used to determine the fate of Ag-NPs after nebulization in terms of agglomeration, internalization and cell attachment. In Figure 3A and B the upper layer of the triple cell co-culture is shown in an overview. The illustrated cells were exposed to the higher Ag-NP dose (278 ng/cm^2^), and were fixed and prepared for TEM after 24 h post-exposure. To reduce misinterpretation due to staining artefacts [43], the cells were treated with uranyl acetate only but without lead citrate. The epithelial cell layer can be seen as well as the porous supporting membrane in the lower left corner. As visible in the close-up view (Figure 3A’ and B’) agglomerated Ag-NPs were observed in vesicles and multi-lamellar bodies inside cells. Single particles inside the cells could not be detected. Furthermore, particles were not detected in other subcellular compartments, attached to the cell membrane or in the nucleus.

### Cytotoxicity

Effects of Ag-NPs and Ag-ions on cell integrity were assessed by measuring the activity of lactate dehydrogenase (LDH) released into the cell culture medium of the lower trans-well chamber. Potential effects were also measured in cultures incubated with lipopolysaccharide (LPS) and tumor necrosis factor alpha (TNF-α), which was used to study possible aggravation effects of Ag-NPs in response to a pro-inflammatory stimulus and the positive control for interleukine-8 (IL-8) release respectively.

**Figure 4:**
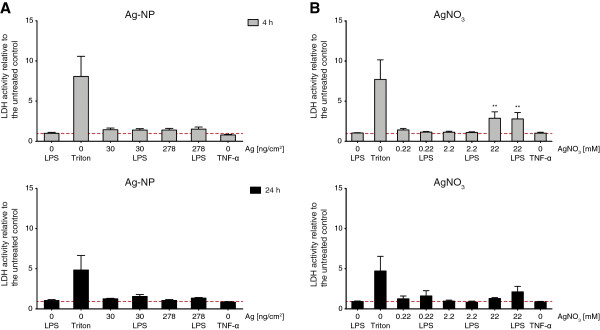
**Cytotoxicity upon Ag-NP and AgNO**_**3 **_**exposure.** Cell integrity as estimated by quantification of extracellular LDH release relative to the unexposed and untreated control (red dashed line) was measured 4 h (grey bars) and 24 h (black bars) after exposure. Cells were exposed to 30 ng/cm^2^ and 270 ng/cm^2^ Ag-NP (**A**) and equal concentrations of 0.22 mM and 2.2 mM as well as 22 mM AgNO_3_ (**B**). As positive control (Triton) cells were treated with Triton X-100 for 4 h and 24 h. LDH release was also measured for cells treated with TNF-α. Error bars represent the standard error of the mean (SEM) for at least 3 independent experiments. A two-way analysis of variance (ANOVA) with a subsequent Bonferroni *post-hoc* test was performed. Values were considered significantly different compared to the unexposed and untreated control with p < 0.01 (**).

Cells were exposed to two different Ag-NP concentrations 1× (30 ng/cm^2^) and 10× (278 ng/cm^2^). To compare the effects of Ag-NPs with Ag-ions, 1×, 10× and 100× AgNO_3_-solutions were prepared with the same concentration as the Ag-NP suspensions (0.22 mM, 2.2 mM and 22 mM) before nebulization. Cells unexposed to Ag-NPs or AgNO_3_ were used as negative controls to calculate relative changes in LDH activity. The reference point (Value = 1) is indicated as dashed red line (Figure 4). Cells lysed with Triton X-100 (TX-100) as positive control revealed the maximum LDH release. Deposition of 30 and 278 ng/cm^2^ Ag-NPs did not significantly increase the LDH activity 4 h and 24 h after exposure (Figure 4A). Similar effects were observed with equal concentrations of AgNO_3_ (0.22 and 2.2 mM), whereas after exposure of 22 mM AgNO_3_ a significant LDH release for LPS untreated (2.88 ± 0.8 fold) as well as for treated cells (2.80 ± 0.80 fold) was monitored 4 h after exposure to Ag-ions (Figure 4B). However, 24 h after exposure of 22 mM AgNO_3_ no difference of LDH activity to the negative control was observed (Figure 4B). AgNO_3_ exposures to A549 monocultures revealed a similar pattern even though a significant increase of LDH activity could only be observed for LPS treated cells after 4 h (see Additional file 1).

### Cytokine/Chemokine secretion

The immune response of the triple cell co-culture system after exposure to Ag-NPs or AgNO_3_ was measured by quantifying the amount of specifically released pro-inflammatory proteins TNF-α and IL-8 via enzyme-linked immunosorbent assay (ELISA) 4 h and 24 h after exposure. Unexposed cells served as negative control. Moreover, cells were also pre-treated with 1 μg/mL LPS 2 h before exposure to study Ag-NP and Ag-ion effects under inflammatory conditions. As positive control, unexposed cells pre-treated with LPS were used. As a positive control for IL-8 secretion, cells were treated with 15 ng/mL TNF-α.

**Figure 5:**
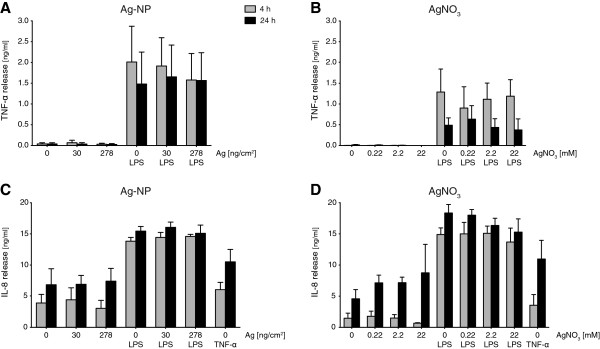
**Protein secretion of pro-inflammatory cytokines TNF-α and IL-8.** The extracellular release of pro-inflammatory markers (ng/mL) TNF-α (**A** and **B**) and IL-8 (**C** and **D**) were analysed by ELISA 4 h (grey bars) and 24 h (black bars) after exposure. Error bars represent the SEM for at least 3 independent experiments.

After 4 h and 24 h, secretion of TNF-α could not be detected when cells were exposed to Ag-NPs (Figure 5A). Following stimulation with LPS, the released TNF-α concentrations of unexposed cells increased (4 h: 2.0 ± 0.9 ng/mL; 24 h: 1.5 ± 0.8 ng/mL). No additive effects were observed following exposure of Ag-NPs to LPS treated cell cultures. Identical to Ag-NP exposures, Ag-ions did not induce an increased TNF-α release (Figure 5B). LPS treated unexposed cells showed an increase of TNF-α secretion (4 h: 1.3 ± 0.6 ng/mL; 24 h: 0.6 ± 0.2 ng/mL). No additive effects were observed following AgNO_3_ exposure of LPS treated cells. The average TNF-α concentrations were lower compared to the levels in the Ag-NP experiments. However, this was not significant and considered to be related to environmental factors such as culture conditions or immune cell activity, which can be very different because of the use of primary cells.

Similar to TNF-α release, IL-8 concentrations for unexposed cells (4 h: 3.9 ± 1.4 ng/mL; 24 h: 6.8 ± 2.6 ng/mL) increased upon LPS treatment (4 h: 13.8 ± 0.6 ng/mL; 24 h: 15.5 ± 0.7 ng/mL) (Figure 5C). Ag-NP exposure did not significantly change the IL-8 release. Incubation with TNF-α stimulated IL-8 secretion to a lesser extent than LPS but showed a similar pattern (4 h: 6.1 ± 1.2 ng/mL; 24 h: 10.5 ± 2.0 ng/mL). Comparable to Ag-NPs, released IL-8 concentrations after AgNO_3_ exposures were not significantly different from unexposed cells (4 h: 1.5 ± 0.8 ng/mL; 24 h: 18.4 ± 1.4 ng/mL) and LPS treated un\exposed cells (4 h: 14.9 ± 1.1 ng/mL; 24 h: 18.4 ± 1.4 ng/mL) (Figure 5D). As for Ag-NP experiments, stimulation with TNF-α revealed a similar but lower concentration pattern (4 h: 3.6 ± 1.7 ng/mL; 24 h: 11.0 ± 3.0 ng/mL) compared to LPS stimulation.

### Real-time reverse transcriptase polymerase chain reaction (real-time RT-PCR) of pro-inflammatory and oxidative stress markers

**Figure 6:**
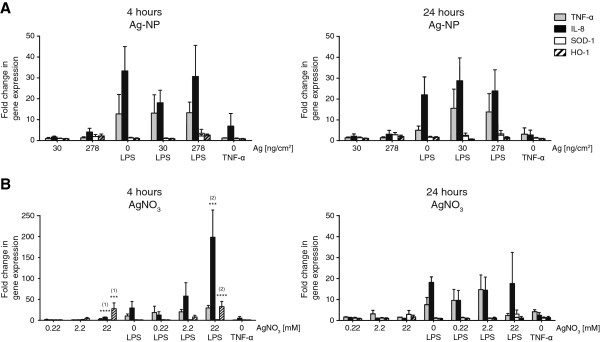
**Quantitative gene expression of pro-inflammatory and oxidative stress markers.** Exposed cells were harvested 4 h and 24 h after exposure and mRNA levels of pro-inflammatory markers *TNF-α* (grey) and *IL-8* (black) as well as oxidative stress markers *SOD-1* (white) and *HMOX-1* (striated) were analysed by real-time RT-PCR. Fold changes of gene expression compared to unexposed untreated controls were calculated with 2^-ΔΔCt^. Error bars represent the SEM for at least 3 independent experiments. A two-way ANOVA with a subsequent Bonferroni *post-hoc* test was performed. Values were considered significantly different compared to unexposed untreated control (1) and unexposed LPS treated control (2) with p < 0.001 (***) or p < 0.0001 (****).

To further study the pro-inflammatory as well as the oxidative stress response of the triple cell co-culture system upon Ag-NP and AgNO_3_ exposure the total RNA of cells 4 h and 24 h after exposure was collected. The relative mRNA induction of the two pro-inflammatory marker genes *TNF-α* and *IL-8* as well as two oxidative stress markers, superoxide dismutase 1 (*SOD-1*) and heme oxygenase 1 (*HMOX-1*) were analysed. To induce inflammatory conditions the cells were treated 2 h before exposure with 1 μg/mL LPS. Fold changes of induction (2^-ΔΔCt^) were calculated according to [44]. The expression of the pro-inflammatory markers *TNF-α* and *IL-8* was not induced for both NP concentrations used after 4 h and 24 h (Figure 6A), which is in agreement with the ELISA results. Upon LPS treatment, expression of *TNF-α* (4 h: 12.8 ± 9.3 fold; 24 h: 5.0 ± 2.0 fold) and of *IL-8* (4 h: 33.3 ± 11.7 fold; 24 h: 21.9 ± 8.6 fold) increased for unexposed cells. Statistically significant differences of *TNF-α* and *IL-8* expression levels upon Ag-NP exposures could not be observed. Expression for the oxidative stress markers *SOD-1* and *HMOX-1* did also not change for any of the conditions assessed. TNF-α treatment induced *IL-8* expression (4 h: 6.9 ± 6.1 fold; 24 h: 2.7 ± 2.4 fold) to a lesser extent compared to LPS treatment. Furthermore, a moderate induction of *TNF-α* after 24 h could be detected (3.1 ± 3.0 fold).

Similar effects could be observed for *TNF-α* and *IL-8* expression upon 0.22 mM and 2.2 mM AgNO_3_-exposures (Figure 6B). However, after 22 mM AgNO_3_ exposure the *IL-8* expression was significantly increased after 4 h (7.3 ± 0.6 fold) which could not any more be observed after 24 h. Furthermore, 22 mM AgNO_3_ exposure following LPS treatment significantly increased *IL-8* gene expression after 4 h (198.5 ± 53.8 fold) compared to unexposed LPS treated cells. An elevated change of expression could also be observed for 2.2 mM AgNO_3_-exposed LPS treated cells after 4 h (57.7 ± 32.1 fold); however, due to high standard deviation this was not significant. Compared to unexposed cells *TNF-α* expression revealed no statistical differences. In contrast to the Ag-NPs, the AgNO_3_-exposures showed a concentration dependent effect of *HMOX-1* expression after 4 h with a significant difference at the highest concentration of 22 mM AgNO_3_ (4 h: 27.7 ± 13.8 fold; 4 h LPS: 32.7 ± 12.3 fold), which diminished after 24 h. TNF-α treatment induced *IL-8* expression (4 h: 5.1 ± 3.9 fold; 24 h: 2.5 ± 1.3 fold) to a lesser extent compared to LPS treatment. Furthermore, a moderate induction of *TNF-α* after 24 h could be detected (4.2 ± 1.0 fold).

### Epithelial monocultures

Since AgNO_3_ exposures have shown both cytotoxic as well as pro-inflammatory effects at the highest concentration used with the triple cell co-cultures, a select number of experiments were repeated with A549 monocultures to compare the two cell culture systems. For the cytotoxicity assay similar results as for the triple cell co-cultures were obtained for A549 monocultures exposed to AgNO_3_ even though significant LDH release could only be observed for LPS treated cells (see Additional file 1). Additionally, release of IL-8 in A549 monocultures exposed to AgNO_3_ could not be detected, even not for LPS treated cells, as A549 do not have a receptor for this endotoxin (see Additional file 2). However, upon stimulation with TNF-α a strong release of IL-8 could be observed.

## Discussion

The aim of the study was to analyse the cytotoxic and pro-inflammatory effects of 20 nm citrate-coated Ag-NPs exposed at the air-liquid interface to an epithelial airway model of the human lung *in vitro*. The study was designed according to a recent publication with 15 nm citrate-coated gold NPs using the same air-liquid cell exposure system and analysis of the same cellular reaction endpoints [41]. Briefly, gold NPs were found to enter the cell in a concentration dependent manner [42] but did not induce oxidative stress nor a pro-inflammatory response. In addition, no synergistic or suppressive effects of the gold NPs could be observed, when simulating an inflammatory environment by LPS. The Ag-NP distribution was homogenous as observed for the gold NPs, however, imaging with TEM revealed a higher agglomeration state inside cellular vesicles for Ag-NPs than for gold NPs. In contrast to gold NP uptake no single Ag-NPs could be detected inside cells. These results were not quantitative as it is assumed that Ag-NPs can also dissolve inside cells.

Ag-NPs did not induce any cytotoxic reactions as could be observed for gold NPs too. The secretion of pro-inflammatory markers did not alter when measured by ELISA and transcriptional induction of pro-inflammatory and oxidative stress markers could not be observed. Furthermore, RT-PCR results confirm the ELISA experiments, as Ag-NPs do not alter the expression of *TNF-α* and *IL-8* under inflammatory conditions.

There is an on-going debate whether Ag-NPs themselves or the ions released from the NPs are responsible for the observed effects [3]. Therefore, we compared the effects when cells were exposed to Ag-NPs and Ag-ions at the same molecular mass by using a sophisticated application. The current study employed the ALICE system to nebulize a defined water-based solution onto cells. Previously, using the ALICE system Lenz et al. compared submerged and air-liquid interface exposures for zinc oxide NPs [40]. The authors found with dose-response measurements significant differences in mRNA expression of pro-inflammatory (*IL-8*) and oxidative stress (*HMOX-1*) markers. Furthermore, Raemy et al. compared aerosol and suspension exposures and found that both exposure strategies differ fundamentally in their dose-response pattern [45]. Both studies emphasize that interactions of NPs with cells depend on the exposure method. Therefore, a direct comparison of the related effects of either NPs or ions negates the problem that ions can be released from the NPs when in suspension, such as in submerged cultures. Thus, AgNO_3_ solutions with the same silver amount as the Ag-NP suspensions were prepared to directly compare particle related to ionic effects at the air-liquid interface. For the 0.22 and 2.2 mM AgNO_3_ concentrations, which contained the same total amount of silver as the Ag-NP suspensions for nebulization, similar results were observed as for the particle exposures. In contrast to Ag-NP exposures minor differences in response to oxidative stress could be observed. However, only when the AgNO_3_ concentration was increased to 22 mM, which was not possible to prepare for Ag-NP suspensions and also would represent an unrealistic high dose, differences in the expression pattern of analysed markers with RT-PCR could be monitored. A pro-inflammatory reaction upon exposure to this high ion concentration could be detected. Moreover, the expression of the pro-inflammatory marker *IL-8* was significantly increased under inflammatory conditions, leading to an aggravating effect in combination with LPS treatment at 4 h after exposure. A direct interaction of LPS and Ag can be excluded since they have been added in different compartments, i.e. LPS was added in the lower well of the two chamber systems and the particles were nebulized on the cells on the upper side of the insert. If there is an interaction within the cells cannot be answered so far. Furthermore, a strong response to oxidative stress could be directly linked to the high concentration of Ag-ions. Also LDH measurements and LSM revealed that cell integrity is impaired only with the highest silver ion concentration. However, the LDH activity measured might be biased due to enzyme inhibition by silver and low cytotoxic levels could therefore be misinterpreted. Since we could not detect impaired cell morphology by LSM and TEM we had no evidence that the LDH test was affected with lower concentrations. In addition, the observed differences to Ag-NPs and Ag-ion concentrations were short-term and reversible within 24 h. This shows a strong influence of the ionic content of the exposed solution as well as a highly concentration dependent manner of inflammatory response to silver.

Many studies have shown that the toxicity of nano-Ag exposed to cells or animals can be related to the size, the shape, the ion content and the concentration, but the findings are highly controversial. On one hand *in vivo*[36] and *in vitro*[18,19] analysis revealed only minor cytotoxic and inflammatory effects after silver exposure. Furthermore, just recently the authors of a study investigating the effects of Ag NPs at the air-liquid interface found only a negligible cytotoxic and minor inflammatory response [46]. On the other hand Ag-NP toxicity was reported by many others whereas ROS dependent DNA damage, anti-proliferative effects, increase of inflammatory markers and subsequent cytotoxicity was observed *in vitro*[15,17,19,20,27,47] and *in vivo*[35,48,49]. However, the mentioned studies do not allow a clear proposition if the observed effects are particle related or due to the ionic contribution as recently has been investigated [3]. Moreover, any cellular reactions upon Ag-NP exposures might not only be related to the dose but also relevant to the exposed cell type [32]. A differentiation of the mechanism of action under submerged conditions *in vitro* and of cellular effects *in vivo* stays difficult. Furthermore, dissolution of the Ag-NP in the aqueous lining layer and inside cells needs further investigations.

Ag-ions have a greater tendency to strongly interact with thiol groups of vital enzymes and phosphorus-containing bases [50]. Because Ag-NPs have been found attached to the membrane of and internalised in bacteria [5,18], these findings suggest that the antibacterial effect is related to the interaction of Ag with the respiratory chain enzymes, which are directly located at the outer membrane of bacteria and an increase in cell permeability due to a structural changes of the membrane. Also Ag induces free radicals and leads therefore subsequently to membrane and DNA damage [51,52] (for a review see [11]). As shown in our study in response to an increasing dose of AgNO_3_*HMOX-1* is upregulated as an answer to oxidative stress and at the highest concentration can lead to cell damage. Therefore, our results suggest concentration related effects, whereas Ag-ions have immediate access to the cells and can interact with a wide variety of molecules. On the other hand when Ag-NPs, which are applied to the air-liquid interface of the cultured lung cells as monodisperse particles, come in contact with the cells they start to highly aggregate inside endocytotic vesicles as TEM pictures revealed. However, if aggregation already occurs in the aqueous hypophase or inside the vesicles cannot be shown. In addition it is also not possible to show if in the aqueous hypophase or inside the cells ions are released from the Ag-NPs as due to the low pH in endosomes and lysosomes the acidic environment is expected to induce Ag-NP dissolution.

Based on realistic exposure scenarios for silver and titanium, Gangwal et al. [13] recently calculated and recommended the concentrations of NPs used for *in vitro* assays. They found that an exposure scenario of a conservative concentration of 1 mg/m^3^ Ag results in a deposition of 0.061 - 0.15 μg/cm^2^ for 5 - 100 nm particles on the lung surface. Our concentrations with 0.03 and 0.27 μg/cm^2^ represent a realistic scenario for short-term exposures. Even a 10 times higher Ag-ion solution resulting in a calculated deposition of 3 μg/cm^2^ was used to observe effects at unrealistic high dose. Also most of the published studies look at short-term effects as pro-inflammatory, oxidative stress and proliferation markers, as well as DNA-damage over a short period of time, i.e. 24 to 48 h, but the applied concentrations represent lifetime exposure doses they apply at once. Therefore, Ag is added in a single exposure at unrealistic high dose and any observed effect does not necessarily represent realistic events when deposited particles accumulate over time. Also our results reveal that at a post-exposure time of 24 h all markers are decreased again to a basic level, which suggests only short-term effects of Ag. However, further studies are necessary to investigate the interference with biological processes for a chronic exposure scenario as Ag-NPs might continuously release Ag-ions. Ag-NPs cannot be rapidly cleared from the biological system in contrast to Ag-ions and therefore might lead to secondary effects over time.

Not only a realistic dose of NPs but also different cell types such as epithelial cells and immune cells have to be used. Co-culture models are better at simulating the real situation in the lung, than monocultures [53-57]. This is particularly important for toxicological studies including oxidative stress and pro-inflammatory reactions in lung cell culture models upon NP exposure. LDH release could only be observed for the highest Ag-ion concentration in A549 monocultures stimulated with LPS in contrary to the triple cell co-cultures. We could also show that there is no IL-8 release in A549 monocultures upon exposure to LPS since these cells do not express the receptor for this endotoxin. These findings point out the importance to include immune cells if risk assessment of NPs is performed.

## Conclusions

By applying a realistic dose of Ag-NPs at the air-liquid interface of a human epithelial alveolar barrier model no significant cytotoxicity, release and induction of pro-inflammatory mediators was observed. The Ag-NPs were endocytosed and highly aggregated inside vesicular structures but they did not cause cytotoxicity, nor induce the release and expression of oxidative stress and pro-inflammatory markers. For equal AgNO_3_ exposure concentrations similar effects were observed. Furthermore, Ag-NPs as well as Ag-ions did not influence expression and release of pro-inflammatory and oxidative stress markers under inflammatory conditions. Only when the concentration of AgNO_3_ was further increased, the exposures did induce the (temporarily) expression of pro-inflammatory and oxidative stress markers revealing a concentration dependent effect. Our results indicate no acute cytotoxic and pro-inflammatory effects for Ag at a realistic exposure dose. Chronic exposure scenarios, however, might reveal other effects due to a prolonged exposure time. As the mammalian cell is complex, the scientific data revealed by many studies is controversial and there is an urgent need for realistic exposure systems as we used in the present study.

## Methods

### Cell culture

Experiments were carried out with a triple cell co-culture model of the human epithelial airway barrier as described in detail by [39,58,59]. Briefly, A549 cells were cultivated in Roswell Park Memorial Institute (RPMI) 1640 medium (w/25 mM HEPES, w/o L-Glutamine, Gibco, Life Technologies Europe B.V., Zug, Switzerland), supplemented with 1% penicillin G/streptomycin sulphate (P/S; 10,000 units/mL/10,000 μg/mL, Gibco), 1% L-Glutamine (L-Glut; Life Technologies Europe) and 10% foetal bovine serum (FBS; PAA Laboratories, Chemie Brunschwig AG, Basel, Switzerland), subsequently referred to as “RPMI complete medium”. For exposure experiments, cells were seeded in BD Falcon™ cell culture inserts (high pore density PET membranes with a growth area of 4.2 cm^2^ and 3.0 μm pores in diameter; Becton Dickinson AG, Allschwil, Switzerland) placed in BD Falcon™ 6-well tissue culture plates (Becton Dickinson) at a density of 0.5 × 10^6^ cells/mL per insert. Cells were grown to confluence for 5 days under submerged conditions (2 mL RPMI complete medium in the upper and 3 mL in the lower transwell chamber). Peripheral blood monocytes were isolated from buffy coats (Blood donation service SRK Bern AG, Switzerland) and cultured in RPMI 1640 supplemented with 5% human serum (HS; Blood donation service), 1% P/S and 1% L-Glut, referred to as “isolation medium”. For the generation of monocyte-derived dendritic cells (MDDCs), the monocytes were cultured for 7 d in isolation medium with additional supplementation of 34 ng/mL IL-4 (R&D Systems Europe Ltd., Abingdon, UK) and 50 ng/mL GM-CSF (R&D Systems), whereas the monocyte-derived macrophages (MDMs) were obtained without any additional supplements for 7 d.

The triple cell co-cultures were set together as described in detail [60] by adding 500 μL of a MDM suspension to the apical and 300 μL of a MDDC suspension to the basal side of the insert. After cultivation for 24 h in the incubator the cells were transferred from submerged to air-liquid interface conditions. The cell culture medium from the upper transwell chamber was removed and the cell culture medium in the lower transwell chamber was replaced by 1.2 mL of fresh isolation medium. After additional 24 h in the incubator at the air-liquid interface, the co-cultures were ready for exposure. In some of the experiments, an inflammatory environment was created by adding 1 μg/mL lipopolysaccharide (LPS) (Pseudomonas aeruginosa, Sigma Aldrich Chemie GmbH, Buchs, Switzerland) into the medium of the lower transwell chamber 2 h before Ag-NP or AgNO_3_ exposure [41].

### Exposure system

Cells were exposed to nanoparticles using the air-liquid interface cell exposure system (ALICE) as previously described by [40,41]. Briefly, the ALICE consists of four main components: a droplet generator (nebulizer), an exposure chamber, a flow system with an incubation chamber providing temperature and humidity conditions suitable for cell cultivation and a quartz crystal microbalance (QCM; Stanford Research Systems, GMP SA, Renens, Switzerland) for real-time measurement of the cell-delivered NP dose. A dense cloud of micron-sized droplets is generated by nebulization of 1 mL Ag-NP suspension using a vibrating membrane droplet generator (investigational eFlow, PARI Pharma GmbH, Munich, Germany). The dense cloud of droplets generated by the eFlow nebulizer is transported at a flow rate of 5 L/min into the exposure chamber (20 × 20 × 30 cm) where it gently deposits onto cells cultured at the air-liquid interface in standard cell culture plates. Droplet deposition occurs due to single particle sedimentation and an effect known as cloud settling, i.e. the cloud of droplets moves like a bulk object rather than a collection of individual droplets [40]. The flow rate is chosen so that the cloud is diverted to all sides by the ground plate of the exposure chamber to form an almost symmetric pattern of vortices providing gentle but sufficient mixing to result in uniform special droplet deposition on the cells. Following the exposure, taking about 15 min, the cells were kept under air-liquid interface conditions for post-exposure incubation times of 4 and 24 h in 5% CO_2_ humidified atmosphere at 37°C.

### Silver nanoparticles

Commercially available 20 nm citrate-coated colloidal Ag-NPs suspended in H_2_O from British Biocell International (EM.SC20; Plano GmbH, Wetzlar, Germany) were used. Measurements by ICP-MS revealed that the NP suspension had a nominal concentration of 0.0006% silver, corresponding to a silver mass concentration of 6 μg/mL. A 4- and 40-fold concentration of colloidal silver nanoparticles was achieved by ultrafiltration using 30 kDa MWCO centrifugal filter units (Vivaspin 20; Sartorius Stedim AG, Tagelswangen, Switzerland) at 3000 × g by diafiltration for 10 min. The Ag-NP solutions were always freshly prepared by ultrafiltration before exposure.

### Nanoparticle characterization

The size distribution and zeta potential of the Ag-NP stock solutions were analysed by dynamic light scattering (Zetasizer Nano Series, Malvern Instruments Ltd., Worcestershire, UK and 90plus Particle Size Analyser with BI-ZTU Autotitrator, Brookhaven Instruments Corp., Holtsville, USA). Images from transmission electron microscopy (TEM) grids exposed to particles in the ALICE during experiments were taken by TEM (Philips CM12, FEI Co. Philips Electron Optics, Zurich, Switzerland) and further analysed by ImageJ particle analyser.

Analysis of dissolved silver was performed by ultrafiltration using Ultracel 3 k Centrifugal Filter Devices (Amicon; Millipore, Zug, Switzerland) with a MWCO of 3 kDa (pore size < 2 nm) as described by [30]. The filtrates were acidified and the total Ag concentration (isotope ^109^Ag) was measured by ICP-MS (Element 2 High Resolution Sector Field ICP-MS; Thermo Finnigan AG, Allschwil, Switzerland).

The silver mass deposited on the cells was determined by ICP-MS as described by [30]. Briefly, exposed silver NPs were collected in 1 mL ddH_2_O placed in empty 6-wells of tissue culture plates. The total Ag concentration (isotope ^109^Ag) in the silver NP suspensions was measured by ICP-MS after digestion with 4 mL of 65% HNO_3_ and 1 mL of 30% H_2_O_2_ in a high-performance microwave digestion unit (MLS 1200 mega, MLS GmbH, Leutkirch, Germany) at a maximal temperature of 195°C. The reliability of the measurements was determined using specific water references (National Water Research Institute, Burlington, Canada). The recovery for silver NPs was 94.7 ± 4.8%.

### Real-time reverse-transcriptase polymerase chain reaction (real-time RT-PCR)

Following post-incubation of Ag-NP and AgNO_3_ exposures for 4 h and 24 h, the insert membranes were cut out, transferred immediately into RNAprotect cell reagent (Qiagen AG, Hombrechtikon, Switzerland) and stored at 4°C until further processing. Cells were detached by vortexing and lysed by centrifuging with QIAshredder columns (Qiagen). Total RNA was isolated using the RNeasy plus kit (Qiagen) according to the manufacturer’s guidelines and RNA concentration was determined by a NanoDrop 2000 (Thermo Scientific, Witec AG, Littau, Switzerland). Reverse transcription (incubation 1 h at 37°C) was carried out with the Omniscript Reverse Transcription Kit (Qiagen) in 10 μL volume with 0.25 μg RNA/reaction, using a master mix consisting of 0.25 mM of each dNTP (Qiagen), 0.5 μM Oligo-dT primers (Qiagen), 10 units RNase inhibitor (RNasin Plus RNase Inhibitor, Promega AG, Dübendorf, Switzerland), 2 units Omniscript Reverse Transcriptase (Qiagen) and 1x buffer RT (Qiagen). Real-time PCR was performed in a reaction volume of 10 μL, with a total of 2 μL of tenfold diluted cDNA, using a Fast SYBR Green master mix (Applied Biosystems, Life Technologies Europe B.V., Zug, Switzerland) with a 50 nM primer mix in a 7500 Fast real-time PCR system (Applied Biosystems). Settings: Denature 20 sec at 95°C, PCR Cycles (40): 3 sec at 95°C, 30 sec at 60°C. Relative expression levels were calculated using the ^ΔΔ^Ct method as described elsewhere [44] with glyceraldehyde-3-phosphate reductase (*GAPDH*) [GenBank: NC_000012] as internal reference gene. The expression levels of heme-oxygenase 1 (*HMOX-1*) [GenBank: CP002685], superoxide dismutase 1 (*SOD-1*) [GenBank: NM_000454], interleukin-8 (*IL-8*) [GenBank: NM_000584] and tumour necrosis factor-α (*TNF-α*) [GenBank: NM_000594] were determined. Primer sequences (Microsynth AG, Balgach, Switzerland) were the following: *GAPDH*: forward 5’- AAC AGC CTC AAG ATC ATC AGC-3’, reverse 5’- GGA TGA TGT TCT GGA GAG CC-3’; *HMOX-1*: forward 5’- TTC TCC GAT GGG TCC TTA CAC T-3’, reverse 5’- GGC ATA AAG CCC TAC AGC AAC T-3’; *SOD-1*: forward 5’- GTG CAG GTC CTC ACT TTA AT-3’, reverse 5’- CTT TGT CAG CAG TCA CAT TG-3’; *IL-8*: forward 5’- CTG GCC GTG GCT CTC TTG-3’, reverse 5’- CCT TGG CAA AAC TGC ACC TT-3’; *TNF-α*: forward 5’- CCC AGG GAC CTC TCT CTA ATC A -3’, reverse 5’- GCT ACA GGC TTG TCA CTC GG -3’.

### Lactate dehydrogenase release

As a general measure for cytotoxicity, the release of lactate dehydrogenase (LDH) from destroyed cells was assessed. For that, the medium of the lower transwell was collected 4 h and 24 h after exposure and stored at 4°C for analysis. The LDH cytotoxicity detection kit (Roche Applied Science, Mannheim, Germany) was used according to the supplier’s manual. LDH was quantified photometrically by measuring at 490 nm, with 630 nm as reference wavelength. Each sample was assessed in triplicate. The values were expressed as fold increase related to the incubator control at appropriate post-exposure times. For positive controls co-cultures were exposed to 0.2% Triton X-100 detergent in H_2_O at 37°C for the same duration as samples were post-incubated.

### Chemokine / Cytokine quantification

Protein release of pro-inflammatory mediators IL-8 and TNF-α were quantified by a commercially available DuoSet ELISA Development Kit (R&D Systems) according to the manufacturer’s protocol. LPS (1 μg/mL) and TNF-α (15 ng/mL; Sigma-Aldrich) served as positive control for TNF-α and IL-8 induction respectively.

### Laser scanning microscopy

The triple cell co-cultures were fixed on the cell culture insert with 3% paraformaldehyde in phosphate buffered saline (PBS) for 15 min at room temperature and then treated with 0.1 M glycine in PBS for 10 min. Before staining, the cells were permeabilised with 0.2% Triton X-100 in PBS for 15 min at room temperature. The cytoskeleton i.e. actin-filaments of all cells were stained with rhodamine phalloidin 1:100 (R-415; Molecular Probes, Life Technologies Europe B.V., Zug, Switzerland) and DNA was stained with DAPI 1 μg/mL (Sigma Aldrich). Preparations for optical analysis were mounted in Glycergel (DAKO Schweiz AG, Baar, Switzerland). The samples were visualized with an inverted Zeiss laser-scanning microscope (LSM) 710 (Axio Observer.Z1, Lasers: HeNe 633 nm, and Ar 488 nm). Image processing was performed with the 3D multi-channel image processing software IMARIS (Bitplane AG, Zurich, Switzerland). Samples with no fluorescence labelling were used to adjust the background parameters for the stained cells in order to avoid unspecific signals from the Ag-NPs or ions (see Additional file 3).

### Transmission electron microscopy

Intracellular particles were visualized by conventional transmission electron microscopy (TEM). For TEM analysis, the exposed cells on the transwell membrane were fixed with 2.5% glutaraldehyde in 0.03 M potassium phosphate buffer for at least 24 h, subsequently washed with potassium phosphate buffer and post-fixed with 1% osmium tetroxide in sodium cacodylate buffer, washed with maleate buffer, and stained en bloc with 0.5% uranyl acetate in maleate buffer. Afterwards, the cells were dehydrated in ascending ethanol series, and embedded in epon [61]. From the embedded cells, ultrathin sections were cut parallel to the vertical axis of the inserts, mounted on copper grids and stained with uranyl acetate. Imaging was done with a Philips CM12 TEM (FEI Co Philips Electron Optics).

### Statistics

All data are presented as the mean ± standard error of the mean (SEM). Statistical analysis was performed with GraphPad Prism 5 (GraphPad Software Inc., La Jolla, California, USA). A two-way analysis of variance (ANOVA) with a subsequent Bonferroni *post-hoc* test was performed. Values were considered significantly different with p < 0.05 (*), p < 0.01 (**), p < 0.001 (***) or p < 0.0001 (****).

## Competing interests

The authors declare that they have no competing interests.

## Authors’ contributions

FH participated in the design of the study, carried out all the experiments and drafted the manuscript. MJDC participated in the design of the study, accompanied the experimental work intellectually and helped to revise the manuscript. FP was involved in performing the ICP-MS experiments. RB and AF were involved in the planning of the study and helped revise the manuscript. OS was involved in technical advisory of the study and helped revise the manuscript. BRR was the project leader; she was involved in planning the design of the study, has intellectually accompanied the experimental work, made substantial contributions to the analysis and interpretation of the data and has been involved in critically revising the manuscript for important intellectual content. All authors read and approved the final manuscript.

## Supplementary Material

Additional file 1 **Cytotoxicity of A549 monocultures upon AgNO3 exposure.** Relative activity of LDH compared to unexposed untreated control (red dashed line) released to the cell culture medium was measured as a marker for cell integrity 4 h (grey bars) and 24 h (black bars) after exposure. A549 monocultures were exposed to concentrations of 0.22, 2.2 and 22 mM AgNO3. As positive control (Triton) cells were treated with Triton X-100 for 4 h and 24 h. An increase for relative LDH activity could be observed for the highest concentration of AgNO3 and LPS treated cells only. Error bars represent the standard error of the mean (SEM) for at least 3 independent experiments. A two-way ANOVA with a subsequent Bonferroni post-hoc test was performed. Values were considered significantly different to unexposed untreated control with p<0.01 (**). Click here for file

Additional file 2 **Protein secretion of IL-8 upon AgNO3 exposure in A549 monocultures.** The release of the inflammatory marker IL-8 into the cell culture medium was analysed by ELISA 4 h and 24 h after exposure. IL-8 secretion upon AgNO3 exposure was not different when compared to the unexposed control. Furthermore, LPS did not stimulate IL-8 release compared to TNF-α treated cells. Error bars represent the SEM for at least 3 independent experiments. Click here for file

Additional file 3 **LSM staining control.** The image represents an unstained triple cell co-culture exposed to 278 ng/cm2 Ag-NPs fixed at 24 h post-exposure time (scale bar = 15 μm). The image was aquired with the same parameters as the labelled samples in Figure 2. Click here for file
