# Supplementary figures and images for: Exposure of silver-nanoparticles and silver-ions to lung cells in vitro at the air-liquid interface
Source: Part Fibre Toxicol. 2013 Apr 4;10:11. doi: 10.1186/1743-8977-10-11 (PMC3639923; doi:10.1186/1743-8977-10-11)

# A1

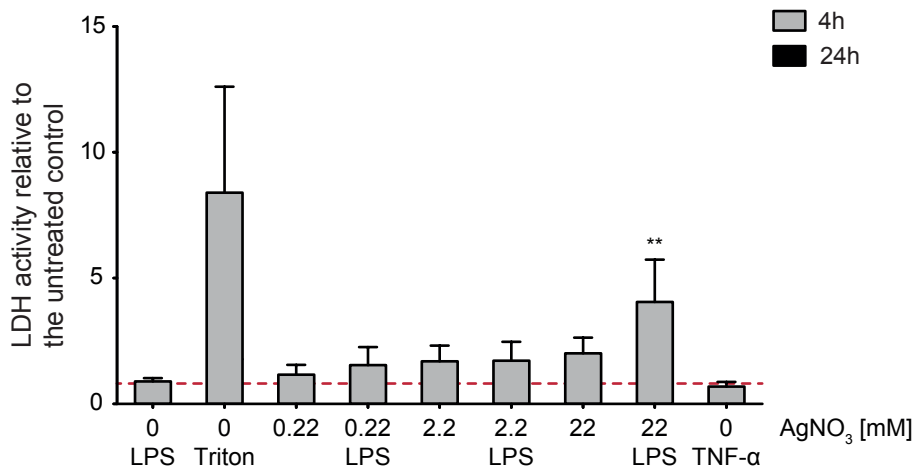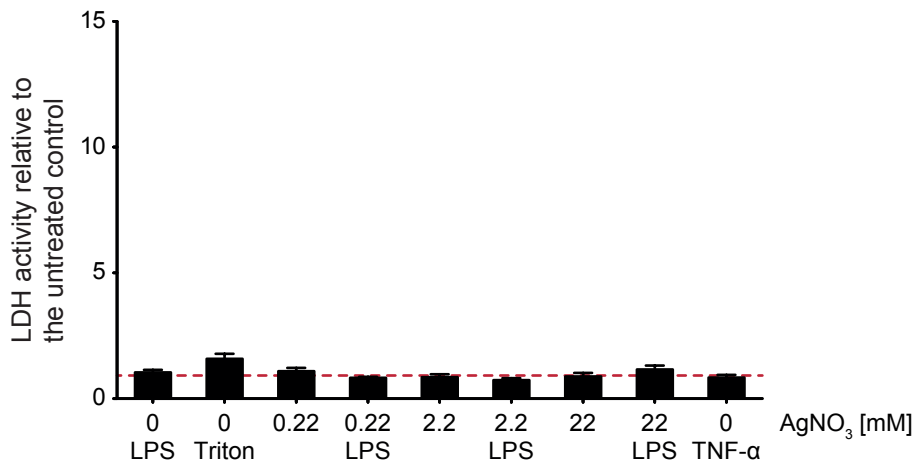

Supplement: Additional file 1 — Cytotoxicity of A549 monocultures upon AgNO3 exposure. Relative activity of LDH compared to unexposed untreated control (red dashed line) released to the cell culture medium was measured as a marker for cell integrity 4 h (grey bars) and 24 h (black bars) after exposure. A549 monocultures were exposed to concentrations of 0.22, 2.2 and 22 mM AgNO3. As positive control (Triton) cells were treated with Triton X-100 for 4 h and 24 h. An increase for relative LDH activity could be observed for the highest concentration of AgNO3 and LPS treated cells only. Error bars represent the standard error of the mean (SEM) for at least 3 independent experiments. A two-way ANOVA with a subsequent Bonferroni post-hoc test was performed. Values were considered significantly different to unexposed untreated control with p<0.01 (**). [file 1743-8977-10-11-S1.pdf]

# A3

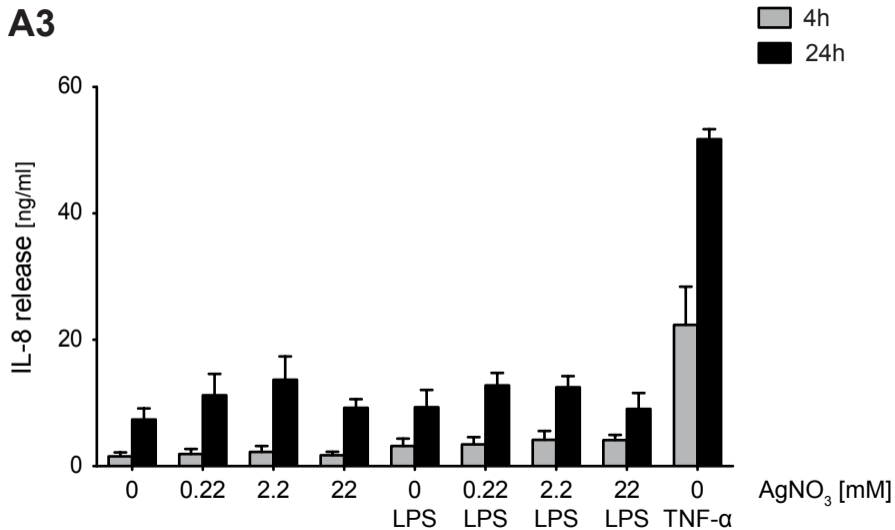

Supplement: Additional file 2 — Protein secretion of IL-8 upon AgNO3 exposure in A549 monocultures. The release of the inflammatory marker IL-8 into the cell culture medium was analysed by ELISA 4 h and 24 h after exposure. IL-8 secretion upon AgNO3 exposure was not different when compared to the unexposed control. Furthermore, LPS did not stimulate IL-8 release compared to TNF-α treated cells. Error bars represent the SEM for at least 3 independent experiments. [file 1743-8977-10-11-S2.pdf]

staining control

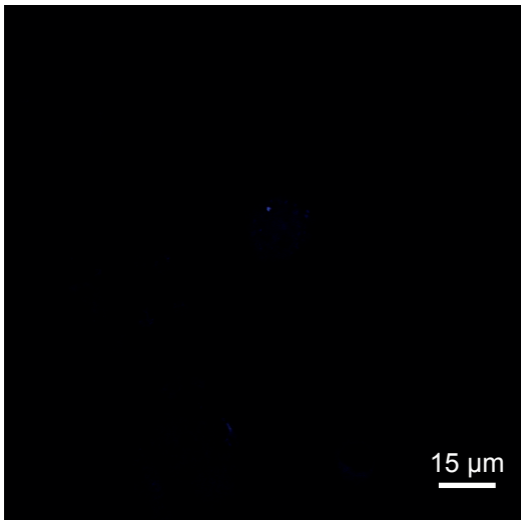

Supplement: Additional file 3 — LSM staining control. The image represents an unstained triple cell co-culture exposed to 278 ng/cm2 Ag-NPs fixed at 24 h post-exposure time (scale bar = 15 μm). The image was aquired with the same parameters as the labelled samples in Figure 2. [file 1743-8977-10-11-S3.pdf]
